# Signaling by adenosine receptors—Homeostatic or allostatic control?

**DOI:** 10.1371/journal.pbio.3000213

**Published:** 2019-04-05

**Authors:** Rodrigo A. Cunha

**Affiliations:** 1 CNC—Center for Neuroscience and Cell Biology, University of Coimbra, Coimbra, Portugal; 2 Faculty of Medicine, University of Coimbra, Coimbra, Portugal

## Abstract

Adenosine modulation is considered both a paracrine signal coordinating different cells in a tissue and a stress signal. Both functions are ensured by 4 types of adenosine receptors (ARs), which have been studied individually. Mice with knockout of all ARs (quad-AR-KO) now allow enquiring the overall function of the adenosine modulation system. The observed “normal” physiology of quad-AR-KO mice indicates that ARs do not regulate homeostasis and are likely recruited to selectively control allostasis.

## Abstract 2

Does the adenosine modulation system control allostasis rather than regulate homeostasis? This Primer explores the implications of a new study of mice that lack all four adenosine receptors.

Adenosine is a central molecule in the life of any cell: it is an intrinsic part of genetics (DNA and RNA), epigenetics (the main methyl donor is S-adenosylmethionine), redox balance (the “A” in NADH [nicotinamide adenine dinucleotide]), and bioenergetic (the “A” in ATP [adenosine 5’-triphosphate]). Not surprisingly, for such an ubiquitous molecule, it has also evolved to become a paracrine signal in eukaryotes to inform cells of metabolic activity (effort or stress): if a cell engages in greater activity or is forced to adapt to stress, more energy (ATP) is consumed, and adenosine is produced and released to the extracellular medium through ubiquitous nucleoside transporters. Most eukaryotic cells and virtually all known mammalian cells are equipped with sensors for adenosine in the form of membrane receptors able to selectively sense extracellular adenosine and to impose alterations of intracellular function. In fact, the 4 known adenosine receptors (ARs; A_1_, A_2A_, A_2B_, and A_3_ receptors) are all metabotropic receptors, i.e., G-protein-coupled receptors that can engage different enzymatic activities and/or alterations of ion channels function to implement metabolic adaptations [1].

After the pioneering efforts of John Daly to identify ARs, a wealth of knowledge has been obtained on the localization and function of the 4 ARs thanks to their cloning and the development of excellent ligands mainly by the efforts of Ken Jacobson’s group [2]. Therefore, tinkering with each subtype of ARs allowed documenting a role for the adenosine modulation system in the control of thermoregulation, cardiac function, adaptive vasodilation, kidney and respiratory functions, inflammation, immunogenic responses to cancer, adaptation to hypoxia, reactivity to stress, locomotion, and memory [1]. This is best heralded by the impact in humans of the consumption of a naturally occurring AR antagonist—caffeine [3].

These 4 ARs have traditionally been divided into 2 families, with A_1_ and A_3_ receptors being considered as inhibitory and A_2A_ and A_2B_ receptors as facilitatory [1]. Although each AR within each of the 2 pairs has unique pharmacological properties (e.g., different sensitivities to adenosine) and different signaling capabilities, ARs are often colocalized in the same cell. This makes it difficult to gauge the overall function of adenosine, because there is always the suspicion that one AR can replace the function of another AR that is selectively manipulated. The novel transgenic mouse (quad-AR-KO mice) developed by Reitman’s team [4], knocking out (KO) the expression of the 4 ARs by cross-breeding transgenic mice with genetic elimination of each AR that were developed by different groups, provides a robust novel tool to address this key question of the overall role of AR signaling in physiology and pathology.

The first important observation was that quad-AR-KO mice display few gross physiological alterations. This seems to argue for a lack of a significant role for AR, either in the control of body temperature, heart rate, blood composition, or acute inflammatory responses. Instead, the observed lack of alteration of basal physiology in quad-AR-KO mice heralds the proposal that the adenosine modulation system is not involved in homeostatic regulation but is essentially devoted to controlling allostasis. In other words, ARs do not participate in the fine-tuning of overall body parameters but are only engaged when the alteration of the environment requires adaptive changes to establish a new homeostatic point for the function of the organism, i.e., when an allostatic response is required. In fact, it was noted that quad-AR-KO mice have a lower life expectancy, probably as a result of the life-long inability to allostatically adapt the organism to a different function of different organs and systems over time. It is worth noting that this observation is in remarkable agreement with the association of coffee consumption with increased lifespan and healthspan in humans [5], although it is still debatable if this effect is associated with caffeine intake (e.g., [6]). Assuming that the benefits afforded by coffee consumption might be due to the intake of its principal component, caffeine [5], this re-enforces the hypothesis that the adenosine modulation system might be of critical importance to the adaptive processes that sustain the functioning of mammals upon ageing.

In the other extreme of life, i.e., during development, the quad-AR-KO mice were reported to growth similarly to control wild-type mice. However, it might still be of interest to detail the role of the AR modulation system during development in this new mouse line. In fact, the pioneering work of Scott Rivkees identified A_1_ receptors as important modulators of the maturation of the heart [7], whereas the correct function of A_2A_ receptors was required for an adequate organization of neuronal networks in the brain [8]. Therefore, in spite of the reported “normal” physiology of quad-AR-KO mice, it would be of interest to refine the evaluation of their early development. This would allow clarifying whether ARs might synergize to mold the development of different organs or whether an eventual abnormal development of different organs might only become functionally evident upon their challenging in disease-like conditions, i.e., when a compressed allostatic capacity is imposed by noxious conditions during adulthood.

The contention that the adenosine modulation system is mostly engaged in allostatic control rather than homeostatic regulation still requires to be directly demonstrated using these quad-AR-KO mice: it will be of interest to confirm if quad-AR-KO mice will respond differently to chronic challenges such as diabetes, hypertension, cancer, or in animal models of neurodegenerative or psychiatric disorders. In fact, the reported observation that quad-AR-KO mice responded similarly to wild-type mice upon acute exposure to lipopolysaccharide (a constituent of gram-negative bacteria sufficient to trigger an inflammatory response), highlights the critical importance of time in the engagement of the adenosine modulation system. This is best heralded by the opposite roles of a particular AR, the A_2A_ receptors, in which their activation dampens acute inflammation, whereas the antagonism of A_2A_ receptors controls the resolution of inflammation and affords benefits in conditions of chronic inflammatory conditions [9]. Actually, the use of the quad-AR-KO mice will be particularly useful for ruling out positive or negative synergic interactions between the different ARs in different conditions, namely, in this observed lack of altered acute inflammation; this is in sharp contrast with the decreased response observed in A_2A_ receptor KO mice that contributed to the concept of A_2A_ receptors as a stop signal of inflammation [10].

The quad-AR-KO mice will also be a useful tool to clarify whether the benefits afforded by coffee and/or caffeine, the most widely consumed psychoactive drug worldwide [11], are only mediated by ARs. First, there is a long-standing debate questioning whether the consumption of coffee might essentially be equivalent to the intake of caffeine, which is considered the major bioactive component of coffee. In fact, epidemiological studies have established that only caffeinated coffee is effective to prevent conditions such as memory deterioration on ageing [12] or depression [13]; this is confirmed by animal studies showing that caffeine and the selective antagonism of A_2A_ receptors is sufficient to prevent memory deterioration in animal models of Alzheimer disease [14] or mood dysfunction upon exposure to repeated stress [15]. However, in other conditions such as diabetes, both caffeinated and decaffeinated coffee provide equieffective benefits [16]. Future exploitation of the quad-AR-KO mice will hopefully help in clarifying this issue. Likewise, the use of this new mouse line will allow establishing whether the effects of caffeine are only mediated by ARs [11], whether there is a threshold of caffeine after which non-AR mechanisms will become preponderant mainly to understand the pathological effects of caffeine [17], or whether some of the long-term effects of caffeine might involve epigenetic alterations associated with metabolic changes [18].

In summary, it is anticipated that this new quad-AR-KO mouse line will allow clarifying several open questions related to the function of the adenosine modulation system, which may provide answers as surprising as the current demonstration that this modulation system mainly controls allostasis rather than regulates homeostasis.

## Abbreviations

AR: adenosine receptor
ATP: adenosine 5’-triphosphate
KO: knockout
NADH: nicotinamide adenine dinucleotide
quad-AR-KO: knockout of all adenosine receptors
